# Disruption of CTCF-YY1–dependent looping of the human papillomavirus genome activates differentiation-induced viral oncogene transcription

**DOI:** 10.1371/journal.pbio.2005752

**Published:** 2018-10-25

**Authors:** Ieisha Pentland, Karen Campos-León, Marius Cotic, Kelli-Jo Davies, C. David Wood, Ian J. Groves, Megan Burley, Nicholas Coleman, Joanne D. Stockton, Boris Noyvert, Andrew D. Beggs, Michelle J. West, Sally Roberts, Joanna L. Parish

**Affiliations:** 1 Institute of Cancer and Genomic Sciences, University of Birmingham, Birmingham, United Kingdom; 2 School of Life Sciences, University of Sussex, Falmer, Brighton, United Kingdom; 3 Department of Pathology, University of Cambridge, Cambridge, United Kingdom; University of Wisconsin-Madison, United States of America

## Abstract

The complex life cycle of oncogenic human papillomavirus (HPV) initiates in undifferentiated basal epithelial keratinocytes where expression of the E6 and E7 oncogenes is restricted. Upon epithelial differentiation, E6/E7 transcription is increased through unknown mechanisms to drive cellular proliferation required to support virus replication. We report that the chromatin-organising CCCTC-binding factor (CTCF) promotes the formation of a chromatin loop in the HPV genome that epigenetically represses viral enhancer activity controlling E6/E7 expression. CTCF-dependent looping is dependent on the expression of the CTCF-associated Yin Yang 1 (YY1) transcription factor and polycomb repressor complex (PRC) recruitment, resulting in trimethylation of histone H3 at lysine 27. We show that viral oncogene up-regulation during cellular differentiation results from YY1 down-regulation, disruption of viral genome looping, and a loss of epigenetic repression of viral enhancer activity. Our data therefore reveal a key role for CTCF-YY1–dependent looping in the HPV life cycle and identify a regulatory mechanism that could be disrupted in HPV carcinogenesis.

## Author summary

Oncogenic human papillomavirus (HPV) infection causes cancers of the anogenital and oropharyngeal tracts. HPV infects undifferentiated basal cells of the epithelium at these body sites and expresses low levels of viral early genes, required for replication of the viral genome. In normal epithelia, cellular migration away from the basal layer induces cell cycle exit and differentiation. However, in an HPV-infected cell, differentiation induces increased transcription of the viral early genes to prevent cell cycle exit, supporting amplification of the viral DNA. In this study, we show that the HPV genome recruits the cellular transcriptional regulators CTCF and YY1, which coordinate an epigenetically repressed chromatin loop between the YY1-bound viral transcriptional enhancer and CTCF-bound early gene region to attenuate early gene expression in undifferentiated cells. As the cells differentiate, YY1 protein expression and recruitment to the viral genome is dramatically reduced. This results in a loss of chromatin loop formation, epigenetic de-repression of the viral genome, and enhanced viral early gene expression. The coordination of viral gene expression with cellular differentiation is vital for persistence of infection and completion of the virus life cycle, and disruption of HPV transcriptional control is also a key step in the development of cancer.

## Introduction

Human papillomaviruses (HPVs) are a family of small, double-stranded DNA viruses that infect epithelia at specific anatomical sites. Infection with any of the 12 mucosal oncogenic HPV types is a risk factor for the development of epithelial cancers such as cancer of the uterine cervix and oropharynx [1]. The majority of these epithelial cancers are caused by infection with the HPV16 and 18 viral subtypes.

The HPV life cycle is dependent on the differentiation of infected keratinocytes. Infection is established in the undifferentiated basal cells of epithelia, allowing the virus access to the cellular DNA replication machinery required to replicate viral episomes. To maintain the cell in a proliferative state, the viral E6 and E7 oncoproteins work synergistically to delay differentiation and prevent cell cycle exit. These essential viral proteins are encoded by transcripts that initiate from a short promoter situated immediately upstream of the early transcription start site, termed P_105_ in HPV18 [2]. The activity of P_105_ is controlled by enhancer and silencer sequences upstream of the promoter in the 850 basepair (bp) viral long control region (LCR). P_105_ contains a canonical TATA box, essential for the recruitment of the general transcription factor II D (TFIID) and the initiation of RNA polymerase II (RNA Pol II)-dependent transcription [3]. Proximal to the TATA box is a keratinocyte-specific 3′ enhancer, which recruits cellular transcription activators such as Sp1 and AP-1 (Fos/Jun) [4–6]. Situated within the 3′ enhancer is a silencer region that contains an array of Yin Yang 1 (YY1) binding sites. YY1 recruitment within this region has strong repressive effects on early gene transcription by the exclusion of AP-1 binding [7,8]. It has also been shown in the related HPV31 that the transcription elongation factor TEF-1 and YY1 work cooperatively to activate a second 5′ distal enhancer within the viral LCR [9], and YY1 binding at this site increases as cells differentiate [10]. However, YY1 binding to the 5′ distal enhancer has minimal effects on transcription in HPV16 [7].

The episomal papillomavirus genome associates with histones to form nucleosomes that are subject to epigenetic modification through the specific recruitment of cellular transcription factors that regulate viral transcription [11]. In HPV31, levels of acetylated histone H3 and H4 within the LCR increased upon cellular differentiation, particularly in the keratinocyte-specific enhancer, and correlated with increased transcription [10].

It is clear that epigenetic regulation of HPV transcription plays an important role in the HPV life cycle and in enhanced viral oncogene expression during disease progression [10,12]; however, the mechanisms involved in this regulation have not been determined. We have previously shown that the chromatin-organising transcriptional insulator protein CCCTC-binding factor (CTCF) associates with oncogenic HPV16 and 18 in the E2 open reading frame (ORF), approximately 3,000 nucleotides downstream of the viral LCR [13]. CTCF is a ubiquitously expressed host-cell chromatin-binding protein that associates with tens of thousands of sites within the human genome [14]. Depending on the context of the binding site, CTCF can function as an epigenetic insulator or coordinate long-range interactions between gene promoters and distant enhancers [15,16]. Notably, mutation of the CTCF binding site within the E2 ORF of HPV18 resulted in increased production of E6/E7 encoding transcripts, leading to hyperproliferation of viral genome–containing keratinocytes in organotypic raft culture [13]. CTCF also binds to the DNA genomes of much larger herpesviruses such as Epstein-Barr virus (EBV), Kaposi sarcoma–associated herpesvirus (KSHV), and herpes simplex virus (HSV-1), and CTCF recruitment in these viruses is important in the regulation of epigenetic silencing of latency-associated genes [17–22]. This regulation is in part brought about by the ability of CTCF to coordinate long-range chromosomal interactions within the viral episomes [19,23]. However, in other contexts, CTCF functions to insulate epigenetic boundaries in these large DNA viruses [18,24].

The mechanism by which CTCF regulates HPV oncogene expression is not known. In this study, we identify CTCF- and YY1-dependent loop formation in the HPV18 genome as the mechanism through which viral oncogene expression is restricted in the early stages of infection in undifferentiated keratinocytes. We show that down-regulation of YY1 following differentiation results in loss of loop formation and reversal of epigenetic silencing and facilitates increased oncogene expression and completion of the viral life cycle.

## Results

### HPV18 induces increased CTCF protein expression in undifferentiated keratinocytes

**Fig 1:**
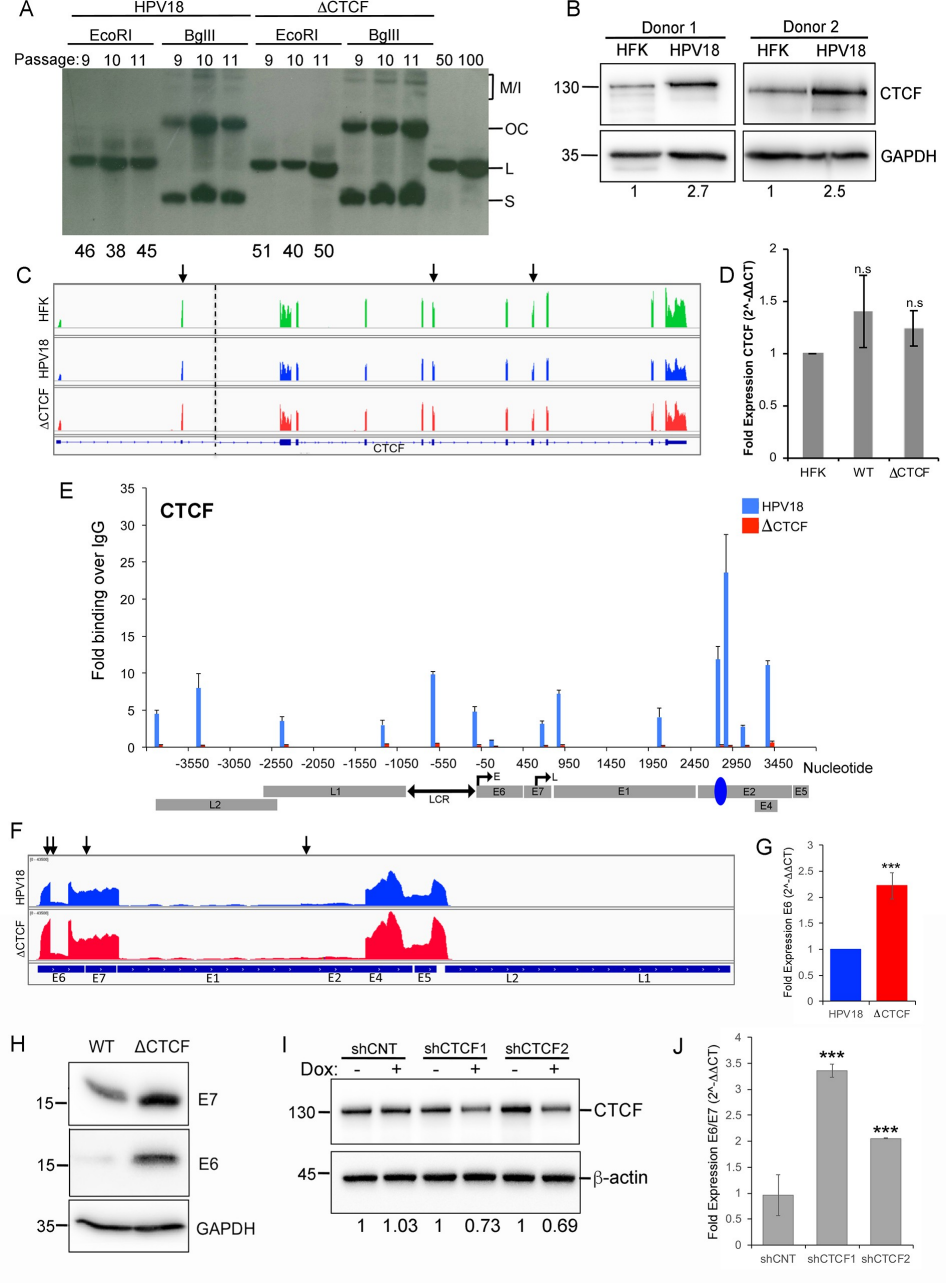
HPV18 recruits CTCF to regulate E6/E7 transcription. Primary HFKs were transfected with HPV18 WT or ΔCTCF genomes and episomes established. **(A)** HPV18 WT and ΔCTCF HFKs were harvested between passages 9 and 11. DNA was digested with *Dpn*I and either *Eco*RI to linearise (L) episomes or *Bgl*II to digest cellular DNA only. The position of multimeric/integrated (M/I), open circle (OC), and supercoiled (S) viral genomes are indicated in addition to copy-number loading controls (50 and 100 copies/cell). The average number of viral episomes per cell was determined in three independent experiments and indicated below the representative blot. **(B)** CTCF protein levels in isogenic HPV18-negative and positive HFKs were assessed by western blotting. CTCF protein was quantified by densitometry and normalised to GAPDH and fold change indicated below. **(C)** CTCF mRNA levels were determined by quantitative RNA-Seq analysis. Image shows the CTCF locus (dotted line indicates a large intronic region truncated in the figure). Data are shown as 0–1,400 counts for each track. Quantification of reads per million counts at the indicated exons (arrows) are shown in S1 Table. (**D**) qRT-PCR quantification of CTCF mRNA using primers that amplify the exon 5/6 junction, normalised to β-actin. Data are shown as mean ± SD. (**E**) CTCF association with the HPV18 genome was determined by ChIP-qPCR in HPV18 WT and ΔCTCF HFKs and fold enrichment of viral DNA with CTCF-specific antibody calculated in comparison to nonspecific FLAG antibody. Data are shown as mean ± SD. **(F)** HPV18 transcripts were analysed by quantitative RNA-Seq. Image shows the HPV18 ORFs and data plotted as 0–43,500 counts for each track. Quantification of reads per million counts at the indicated loci (arrows) are shown in S2 Table. (**G**) qRT-PCR quantification of E6 encoding mRNA using primers that anneal upstream of the E6 splice donor site (nt 131–180) normalised to β-actin. Data are shown as mean ± SD. (**H**) E6 and E7 protein levels in HPV18 WT- and ΔCTCF-genome–containing HFKs were assessed by western blotting. **(I)** CTCF protein depletion by doxycycline-induced (+) shRNA expression. Fold reduction compared to uninduced cells (–) is indicated. CNT shRNA is also shown. **(J)** E6/E7 transcript levels were quantified by qRT-PCR following depletion of CTCF, normalised to β-actin, and shown as fold change over levels in the absence of doxycycline (2^-ΔΔCT^). Data are represented as mean ± SD (***p < 0.001). All raw quantitative data are enclosed in S1 Data. ChIP-qPCR, chromatin immunoprecipitation followed by quantitative PCR; CNT, nontargeting control; CTCF, CCCTC-binding factor; E, early promoter; GAPDH, Glyceraldehyde-3-phosphate dehydrogenase; HFK, human foreskin keratinocyte; HPV, human papillomavirus; IgG, immunoglobulin G; L, linear; M/I, multimeric/integrated; nt, nucleotide; OC, open circle; ORF, open reading frame; qRT-PCR, quantitative reverse transcriptase-PCR; RNA-Seq, RNA-Sequencing; S, supercoiled; shRNA, short hairpin RNA; WT, wild-type.

To analyse the function of CTCF in the life cycle of HPV18, primary human keratinocytes, the natural host cell of HPV, were transfected with religated HPV18 genomes, and replicating episomes were established that were stably maintained at approximately 50 copies per cell. We previously showed that mutation of the CTCF binding site within the E2 ORF of HPV18 (HPV18 ΔCTCF) results in a marked reduction of CTCF binding at this site with no effect on the establishment of HPV18 episomes in primary keratinocytes [13]. However, the long-term persistence of herpesvirus saimiri (HVS) has been shown to be dependent on CTCF [25]. Therefore, we serially passaged HPV18 wild-type (WT)- and ΔCTCF-genome–containing human foreskin keratinocytes (HFKs) and performed Southern blot analysis to examine HPV18 episome copy-number variation over time. The genome copy number at all passages analysed (9–11) was similar between HPV18 WT and ΔCTCF genomes, demonstrating that CTCF binding within the E2 ORF does not play a role in the persistence of HPV18 episomes (Fig 1A). It is important to note that all of the experiments included in this study were performed on cells between passages 9 and 11 to ensure consistent episomal copy number between HPV18 WT and ΔCTCF cultures since viral episomes can integrate into the host genome in long-term culture [26].

To determine whether HPV18 genome establishment alters CTCF protein expression, we quantified CTCF protein in isogenic primary HFKs. We observed a >2.5-fold increase in CTCF protein expression following establishment of HPV18 episomes (Fig 1B). This was consistent in two independent donors and is in agreement with a previous study that demonstrated an increase in CTCF protein expression in HPV31-positive neoplastic cervical keratinocytes compared to HFKs [27]. Interestingly, the HPV18-induced increase in CTCF protein is post-transcriptional since quantitative RNA-Sequencing (RNA-Seq) and quantitative reverse transcriptase-PCR (qRT-PCR) analysis of CTCF transcripts did not show any significant differences in CTCF transcript levels following establishment of HPV18 episomes (Fig 1C and 1D and S1 Table).

### CTCF binding within the E2 ORF represses E6/E7 transcript production

To determine whether abrogation of CTCF binding at the E2 ORF affects CTCF recruitment elsewhere in the viral episome, we performed chromatin immunoprecipitation followed by quantitative PCR (ChIP-qPCR) to specifically amplify CTCF-bound regions throughout the HPV18 genome (Fig 1E). CTCF binding was enriched at the previously identified E2 ORF binding site in cells containing HPV18 WT genomes. In addition, CTCF-enriched regions were identified within the viral LCR, close to the late promoter, and within the L2 ORF. Interestingly, abrogation of CTCF binding at the E2 ORF by mutation resulted in an almost complete loss of CTCF recruitment to all regions of the viral genome, suggesting that CTCF binding at the E2 ORF influences recruitment to regulatory regions that do not contain CTCF binding sites. This phenomenon was consistent in both keratinocyte donors tested.

We previously concluded that CTCF recruitment is important in the regulation of HPV18 oncogene expression in differentiated epithelia [13]. Consistent with these results, we found that in undifferentiated cells, transcripts originating from the early promoter were increased in abundance in quantitative RNA-Seq experiments (Fig 1F and S2 Table), which was confirmed by qRT-PCR (Fig 1G). Importantly, our RNA-Seq analysis showed that this increase in early transcripts is specific to E6/E7 encoding spliced transcripts and not to alternatively spliced E2 encoding mRNA species (Fig 1F and S2 Table), which is in agreement with our previous observation that E2 protein expression is not altered in HPV18 ΔCTCF genomes compared to WT [13]. E6 and E7 protein translated from the polycistronic message increased 11.3- and 1.9-fold, respectively, when the CTCF site was mutated (Fig 1H). To exclude the possibility that abrogation of CTCF binding by mutation of the E2–CTCF binding site results in increased E6/E7 transcription by inadvertently affecting the binding of other factors involved in an alternative regulatory network, CTCF protein levels were depleted by doxycycline-induced expression of two independent CTCF-specific shRNA molecules in HPV18 WT-genome–containing cells (Fig 1I). qRT-PCR analysis of E6/E7 encoding transcript levels demonstrated that partial depletion of CTCF protein resulted in a significant increase in E6/E7 encoding transcripts (Fig 1J). This increase in E6/E7 transcripts was not observed following induction of a nontargeting shRNA control (Fig 1J).

### CTCF reduces chromatin accessibility and epigenetically represses the HPV18 LCR

**Fig 2:**
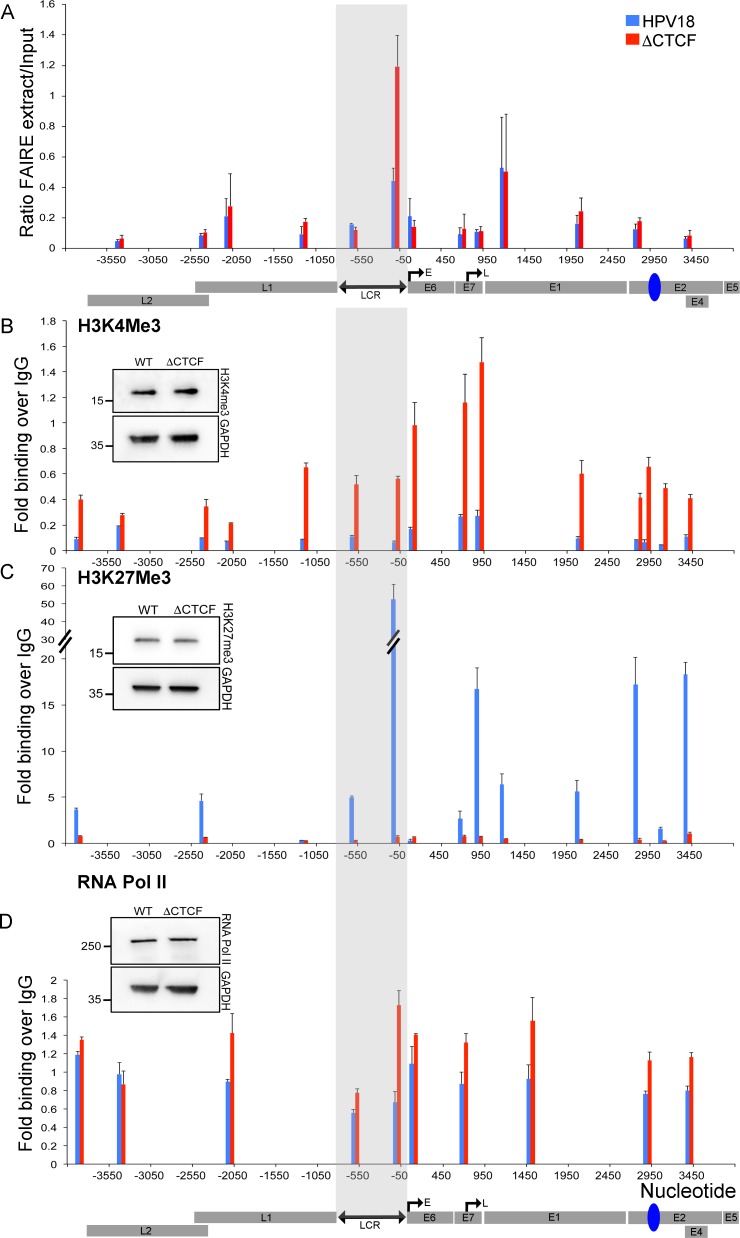
CTCF regulates the topology, epigenetic status, and RNA Pol II recruitment to the HPV18 episome. **(A)** The accessibility of chromatin was assessed by FAIRE. Data show the mean ± SD of qPCR analysis of amplicons in the HPV18 genome of FAIRE-extracted samples compared to input. A higher FAIRE extract/input ratio indicates a more open chromatin structure (***p < 0.001). Enrichment of H3K4Me3 **(B)**, H3K27Me3 **(C)**, and RNA Pol II **(D)** was assessed by ChIP-qPCR. Data show qPCR analysis of ChIP as fold binding over IgG (FLAG antibody) and are represented as mean ± SD. The inset western blot images show relative protein expression in HPV18 WT- and ΔCTCF-genome–containing cells. The linearised HPV18 genome and CTCF–E2 ORF binding site (blue) are indicated below. All raw quantitative data are enclosed in S1 Data. ChIP-qPCR, chromatin immunoprecipitation followed by quantitative PCR; CTCF, CCCTC-binding factor; E, early promoter; FAIRE, formaldehyde-assisted isolation of regulatory elements; GAPDH, Glyceraldehyde-3-phosphate dehydrogenase; H3K4Me3, histone 3 lysine 4 trimethylation; H3K27Me3, histone 3 lysine 27 trimethylation; HPV, human papillomavirus; IgG, immunoglobulin G; L, late promoter; LCR, long control region; ORF, open reading frame; qPCR, quantitative PCR; RNA Pol II, RNA polymerase II; WT, wild-type.

Our data show that recruitment of CTCF within the E2 ORF represses HPV18 early gene expression, and we hypothesised that this was due to repression of early promoter activity. Regulatory genomic elements are depleted of nucleosomes, and the remaining nucleosomes are enriched in active chromatin marks (e.g., acetylated lysine residues in histone H3 and H4) [28]. Formaldehyde-assisted isolation of regulatory elements (FAIRE) can be used to identify open and nucleosome-depleted enhancer regions of DNA [29]. To gain mechanistic insight into the control of HPV early promoter activity by distal CTCF binding, the chromatin accessibility of HPV18 episomes was analysed by FAIRE. We consistently observed a higher FAIRE-to-input amplification ratio, indicative of open chromatin at the HPV18 WT viral enhancer and early promoter (Fig 2A). Notably, there was a significant enrichment of open chromatin at the early promoter of HPV18 ΔCTCF genomes (Fig 2A; *p* < 0.001). This increased chromatin accessibility was consistent between independent donor lines and suggests a mechanism by which CTCF binding at the distal E2 binding site influences nucleosome occupancy within the viral LCR.

Interestingly, we observed that immediately downstream of the open chromatin area in the HPV18 WT genome is a region of closed chromatin in the E6 and E7 ORFs and the late promoter, P_811_. These findings are in agreement with previous DNase I footprinting experiments that demonstrated dynamic nucleosome binding in the viral enhancer and tightly held nucleosomes at the viral late promoter [11]. FAIRE analysis of the HPV18 episome also revealed an area of open chromatin within the E1 ORF, the specific function of which remains unknown.

We next investigated whether the increased accessibility of chromatin within the viral LCR following disruption of CTCF binding was associated with any change in active and repressive epigenetic marks. Using ChIP-qPCR, we analysed levels of the active-promoter–associated H3K4Me3 mark and the polycomb repressor complex (PRC)-associated repressive H3K27Me3 mark across the HPV18 genome. These experiments revealed an enrichment of H3K4Me3 in HPV18 ΔCTCF genomes compared to WT, particularly within the viral enhancer and immediately downstream of the early promoter, indicative of active transcription (Fig 2B). In contrast, enrichment of the repressive H3K27Me3 mark was detected in the enhancer and early gene region of HPV18 WT genomes. This finding was surprising, given that expression of E6/E7 has been shown to cause a global reduction in cellular H3K27Me3 [30]. The enrichment of H3K27Me3 was markedly decreased to almost undetectable levels on HPV18 ΔCTCF genomes (Fig 2C). This epigenetic switching of viral genomes unable to bind CTCF at the E2 ORF is consistent with increased transcriptional activity of the viral early promoter in ΔCTCF genomes and explains the observed alterations in chromatin accessibility identified by FAIRE (Fig 2A).

Since H3K27Me3 has been shown to inhibit recruitment of the general transcription machinery, we examined RNA Pol II recruitment. Indeed, enrichment of RNA Pol II was observed at the early promoter and within the early gene region in HPV18 ΔCTCF compared to WT, consistent with increased transcription levels (Fig 2D).

### Alteration of the chromatin structure in the viral LCR alters host-cell epigenetic modifier recruitment

**Fig 3:**
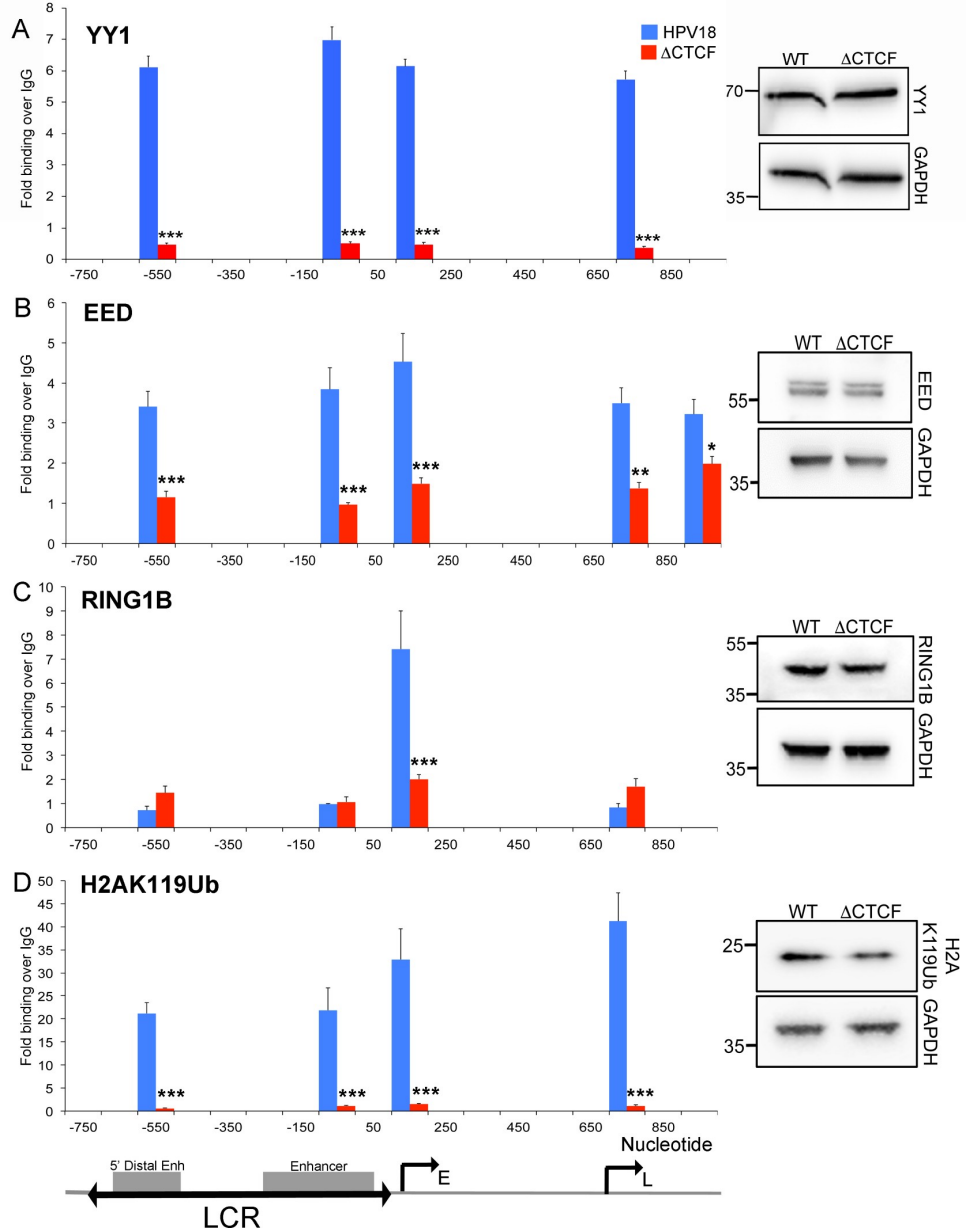
Abrogation of CTCF binding to the E2 ORF reduces YY1 and polycomb recruitment to the HPV18 episome. YY1 **(A),** EED **(B)**, Ring1B **(C),** and H2AK119Ub **(D)** enrichment were assessed by ChIP-qPCR of amplicons within the HPV18 LCR. Data are represented as mean ± SD. (*p < 0.05, **p < 0.01, ***p < 0.001). Relative protein expression is shown in the western blots. The viral LCR, Enh regions, and early and late promoters are indicated. All raw quantitative data are enclosed in S1 Data. ChIP-qPCR, chromatin immunoprecipitation followed by quantitative PCR; CTCF, CCCTC-binding factor; E, early promoter; EED, embryonic ectoderm development; Enh, enhancer; GAPDH, Glyceraldehyde-3-phosphate dehydrogenase; H2AK119Ub, histone 2A lysine 119 ubiquitinylation; HPV, human papillomavirus; L, late promoter; LCR, long control region; ORF, open reading frame; Ring1B, ring finger protein 1B; WT, wild-type; YY1, Yin Yang 1.

Numerous host-cell transcriptional regulators have been shown to specifically bind to the HPV LCR and regulate transcription of viral early genes. To determine whether CTCF influences recruitment of specific cellular regulators of HPV18 transcription, we analysed enrichment of transcription factors that regulate early gene transcription using ChIP-PCR. Our analysis revealed that mutation of the CTCF binding site resulted in significantly reduced binding of the YY1 transcription factor at both the 5′ and 3′ enhancers in the viral LCR and at the early and late promoter regions (Fig 3A). Since YY1 functions in the sequence-specific recruitment of PRCs PRC1 and PRC2, and we detected enrichment of the PRC2-associated H3K27Me3 repressive mark in WT HPV18 genomes, we examined PRC1 and PRC2 binding in the HPV18 genome in WT- and ΔCTCF-genome–containing cells. We found that the PRC2 subunit embryonic ectoderm development (EED) was significantly depleted at the viral enhancer and early promoter in HPV18 ΔCTCF genomes compared to WT, consistent with the observed loss of H3K27Me3 (Fig 3B). Reinforcement of repressed chromatin is achieved via recruitment of PRC1 to the H3K27Me3 mark and ubiquitylation of K119 on histone H2A by the PRC1 E3 ubiquitin ligase, ring finger protein 1B (Ring1B) [31]. Our data demonstrated that Ring1B was associated with the early promoter in HPV18 WT genomes, but binding was dramatically reduced in ΔCTCF genomes (Fig 3C). Loss of Ring1B was coincident with an almost complete loss of histone 2A lysine 119 ubiquitinylation (H2AK119Ub) (Fig 3D). These data indicate that in addition to reduced PRC2 recruitment, PRC1 recruitment to the viral LCR is significantly reduced in HPV18 ΔCTCF episomes. Our data are consistent with a model in which the abrogation of CTCF binding in the E2 ORF results in a loss of YY1 binding to the viral LCR, causing reduced PRC1 and PRC2 recruitment. This leads to reduced H3K27Me3 and H2AK119Ub deposition, de-repression of the HPV18 early promoter, and the up-regulation of viral oncogene expression.

### Epigenetic regulation of HPV18 early gene transcription is mediated by CTCF-YY1–dependent loop formation

**Fig 4:**
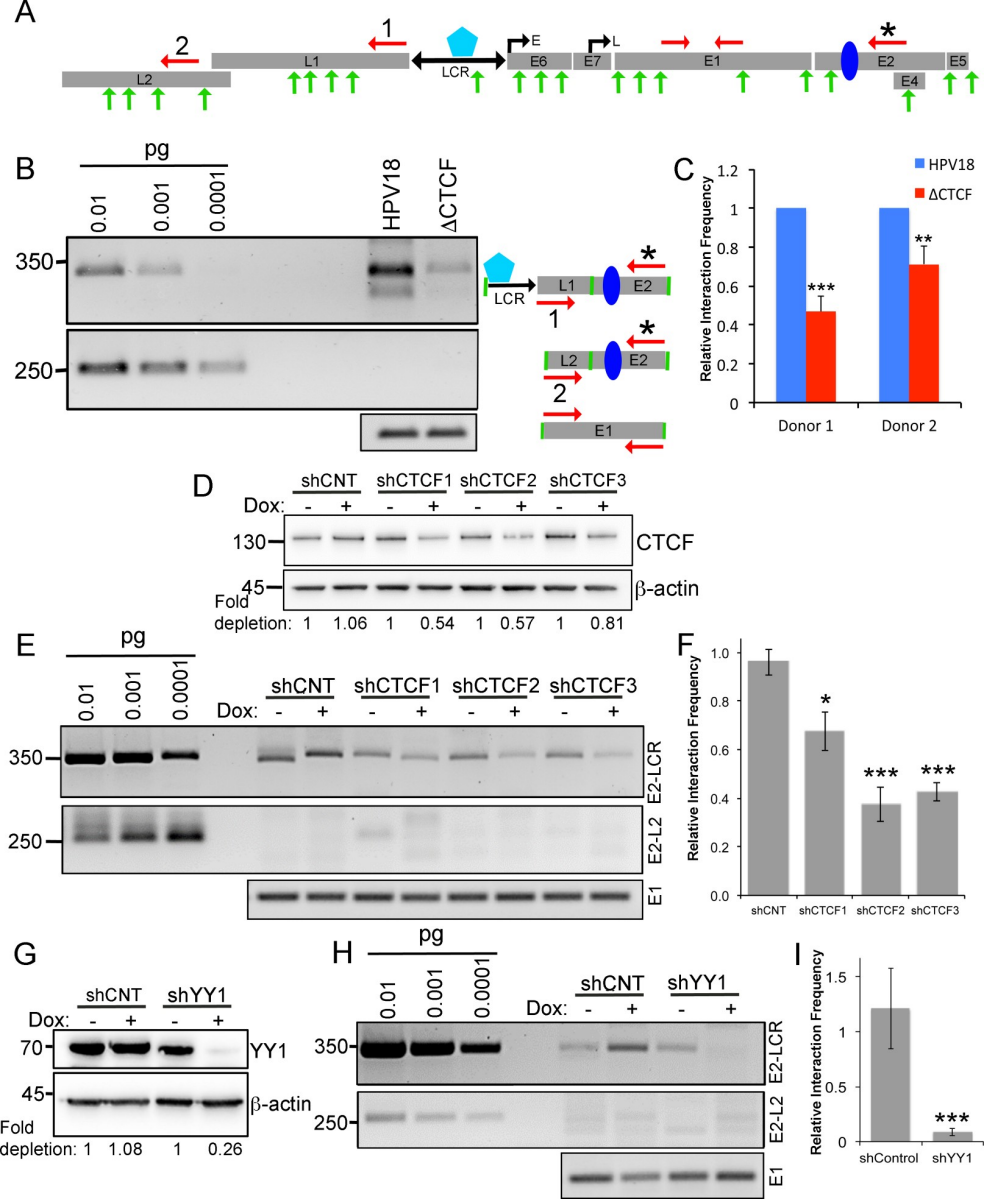
CTCF and YY1 coordinate intrachromosomal interactions within the HPV18 episome between the E2 ORF and the LCR. **(A)** Diagram of the HPV episome is depicted in linear form. ORFs, LCR, early (E) and late (L) promoters, CTCF (blue) and YY1 (aqua) binding sites, *Nla*III restriction sites (green arrows), and primer annealing sites (red arrows) including the E2–ORF anchor primer (*), LCR primer (1), and L2–ORF primer (2). **(B)** PCR analysis of 3C products from HPV18 WT- and ΔCTCF-genome–containing cells as indicated by the schematic to the right. Amplification of serial dilutions (pg) of synthesised predicted ligation products are shown in lanes 1–3, and molecular weight markers are shown on the left. **(C)** Densitometric analysis of 3C products in HPV18 WT- and ΔCTCF-genome–containing cells in two independent keratinocyte donors. Data are the mean ± SEM of at least three independent experiments. **(D)** Efficiency of CTCF depletion relative to control is indicated below the image in induced cells (+) compared to expression in uninduced cells (–). **(E)** 3C analysis was performed following Dox-induced (+) shRNA expression in shCNT and shCTCF (1, 2, and 3) transduced HPV18-genome–containing HFKs. The upper panel shows amplification of E2–LCR ligation product, and the middle panel shows amplification of E2–L2 ligation product. Lower panel shows E1 ORF amplification. **(F)** 3C products were analysed by densitometry, and relative intensity of bands in comparison to uninduced samples was determined. Data are the mean and SEM of at least three independent experiments. **(G)** YY1 depletion was assessed by western blotting alongside β-actin as a loading control. Fold depletion in comparison to uninduced samples is indicated below the blots. **(H)** 3C analysis of E2–LCR interactions following shRNA-mediated depletion of YY1. E2–LCR interactions are shown in the top panel in uninduced (–) and induced (+) HPV18-genome–containing HFKs transduced with shCNT and shYY1 lentivirus. E2–L2 interactions are shown in the bottom panel. **(I)** 3C products were analysed by densitometry, and relative intensity of bands in comparison to uninduced samples was determined. Data are the mean and SEM of six independent experiments. *p < 0.05, **p < 0.01, ***p < 0.001 in all experiments. All raw quantitative data are enclosed in S1 Data. CNT, nontargeting control; CTCF, CCCTC-binding factor; Dox, doxycycline; E, early promoter; HFK, human foreskin keratinocyte; HPV, human papillomavirus; L, late promoter; LCR, long control region; ORF, open reading frame; SEM, standard error of the mean; shRNA, short hairpin RNA; WT, wild-type; YY1, Yin Yang 1; 3C, chromosome conformation capture.

Studies have shown that CTCF and YY1 are able to directly interact and that the assembly of this protein complex induces chromatin loop formation between distant loci [32,33]. We therefore hypothesised that the repressive effects of CTCF binding in the E2 ORF is mediated through loop formation between the YY1-bound viral enhancer and the downstream CTCF-bound E2 ORF. To test this hypothesis, we used chromosome conformation capture (3C), a method that can be used to directly measure inter- and intramolecular interactions between specific distal loci. HPV18 WT and ΔCTCF genomes were cross-linked in situ, and chromatin was extracted and digested with the *Nla*III restriction enzyme, which restricts the viral DNA at multiple sites (Fig 4A). Digestion efficiency was determined for each sample by qPCR analysis of amplicons that are sensitive to digestion compared with a PCR amplicon that is insensitive to digestion. Samples were only processed further if the digestion efficiency was above 90%. Proximity ligation at low dilution was then carried out to ligate restriction fragments containing DNA loci that are physically associated through interactions between chromatin-bound factors. We designed unidirectional PCR primers to amplify a 346 bp amplicon across the ligation junction that would be formed if the restriction fragments containing the CTCF site in the E2 ORF and the LCR were ligated together as a result of the formation of a chromatin loop (Fig 4A and 4B).

3C analysis of HPV18 WT genomes consistently detected a PCR product of the correct size formed by ligation of the E2 ORF to the LCR. In addition, the 346 bp PCR products were excised from the gel and sequenced to confirm ligation between the YY1-bound viral LCR and CTCF-bound E2 ORF (S1 Fig). Given the small size of the HPV genome, we controlled for nonspecific interactions by carrying out PCR using primers designed to amplify ligation products between the CTCF-bound E2 ORF and the L2 ORF. No interactions were detected between these regions of the genome, although the E2–L2 primers efficiently amplified synthesised DNA molecules containing this ligation junction (Fig 4B, middle panel). We also performed a PCR reaction with primers that anneal within the E1 ORF that are insensitive to *Nla*III digestion to ensure equal amplification of digested input chromatin in all 3C experiments (Fig 4B, lower panel). Notably, we found that looping between the YY1-bound LCR and the E2 ORF was significantly reduced in HPV18 ΔCTCF genomes in both donor lines tested (Fig 4C). To confirm that the reduction in E2 ORF–LCR interactions in HPV18 ΔCTCF was due to abrogation of CTCF binding, CTCF protein levels were depleted in HPV18-genome–containing keratinocytes using three independent shRNA lentiviral vectors following induction of shRNA expression with doxycycline (Fig 4D). While E2–LCR interactions were consistently detected in control shRNA-expressing cells, this interaction was significantly reduced following partial depletion of CTCF with all three independent shRNA molecules (Fig 4E and 4F). These data therefore demonstrate that CTCF directs the formation of a chromatin loop between the viral LCR and E2 ORF.

We next assessed the role of YY1 in the formation of this chromatin loop by shRNA-mediated depletion of YY1. HPV18-genome–containing cells were transduced with lentivirus expressing doxycycline-inducible YY1-specific shRNA (Fig 4G). 3C analysis demonstrated that depletion of YY1 resulted in a consistent and significant reduction in E2–LCR interactions (Fig 4H and 4I). Together, our data demonstrate that E2–LCR loop formation in the HPV18 genome requires both CTCF and YY1.

**Fig 5:**
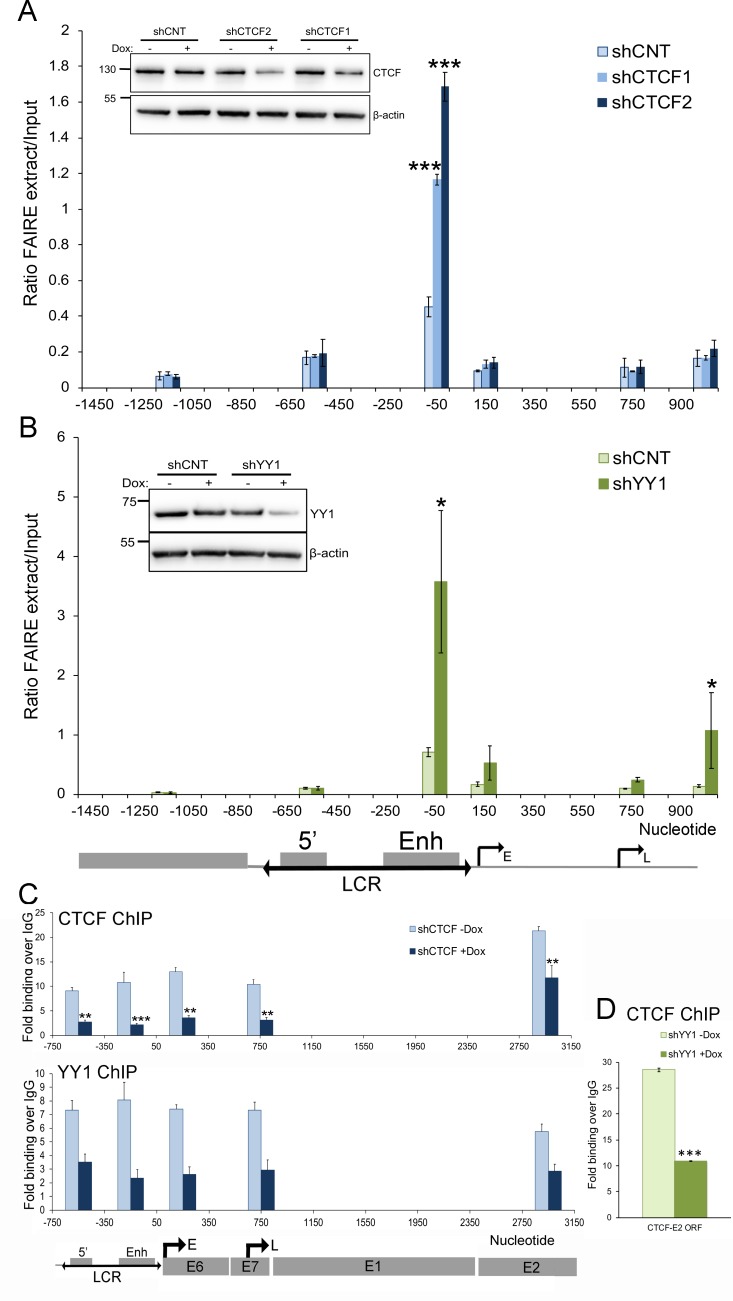
Depletion of CTCF or YY1 results in increased chromatin accessibility within the HPV18 LCR. Chromatin topology was assessed by FAIRE as previously described. Depletion of CTCF (**A**; shCTCF1 and shCTCF2) and YY1 (**B**; shYY1) protein in WT HPV18 genome containing cells is shown in the inset panels in comparison to shCNT. Data show mean ± SD of qPCR analysis of FAIRE-extracted samples of representative experiments performed in triplicate. Each experiment was performed at least three times with similar results. (**C**) Recruitment of CTCF (upper panel) and YY1 (lower panel) to the viral LCR and E2–CTCF binding site following Dox induction of shCTCF was assessed by ChIP-qPCR, and (**D**) recruitment of CTCF to the E2 ORF following shYY1 induction was assessed by ChIP-qPCR. Data show mean ± SD. ***p < 0.001, **p < 0.01, *p < 0.05 in all experiments. All raw quantitative data are enclosed in S1 Data. ChIP-qPCR, chromatin immunoprecipitation followed by quantitative PCR; CTCF, CCCTC-binding factor; Dox, doxycycline; E, early promoter; Enh, enhancer; FAIRE, formaldehyde-assisted isolation of regulatory elements; HPV, human papillomavirus; IgG, immunoglobulin G; L, late promoter; LCR, long control region; ORF, open reading frame; qPCR, quantitative PCR; shCNT, nontargeting shRNA control; shRNA, short hairpin RNA; WT, wild-type; YY1, Yin Yang 1.

To confirm that YY1-CTCF–dependent chromatin loop formation within the HPV18 episome regulates chromatin topology, we used FAIRE to assess the chromatin structure within the viral LCR and flanking regions following shRNA-mediated depletion of CTCF (Fig 5A) and YY1 (Fig 5B). These experiments revealed a significant increase in chromatin accessibility within the LCR following depletion of either CTCF or YY1, consistent with the increase in chromatin accessibility in HPV18 ΔCTCF genomes that are unable to bind CTCF.

Our data show that CTCF and YY1 contribute to chromatin loop formation within the HPV18 genome, resulting in epigenetic repression of early gene expression. To determine whether CTCF and YY1 binding to the viral genome are interdependent, we depleted CTCF protein by shRNA induction and performed ChIP for CTCF and YY1 at the viral LCR and E2–CTCF binding site (Fig 5C). Depletion of CTCF protein resulted in reduced recruitment of CTCF to the E2–CTCF binding site and also a reduction in binding in the viral LCR. Notably, CTCF depletion also resulted in reduced YY1 recruitment to the LCR, and YY1 depletion resulted in reduced CTCF binding at the E2–CTCF binding site (Fig 5D). These data suggest that CTCF binding at the E2 ORF stabilises YY1 binding at the LCR and vice versa and that the enrichment of these proteins within the viral genome is interdependent.

**Fig 6:**
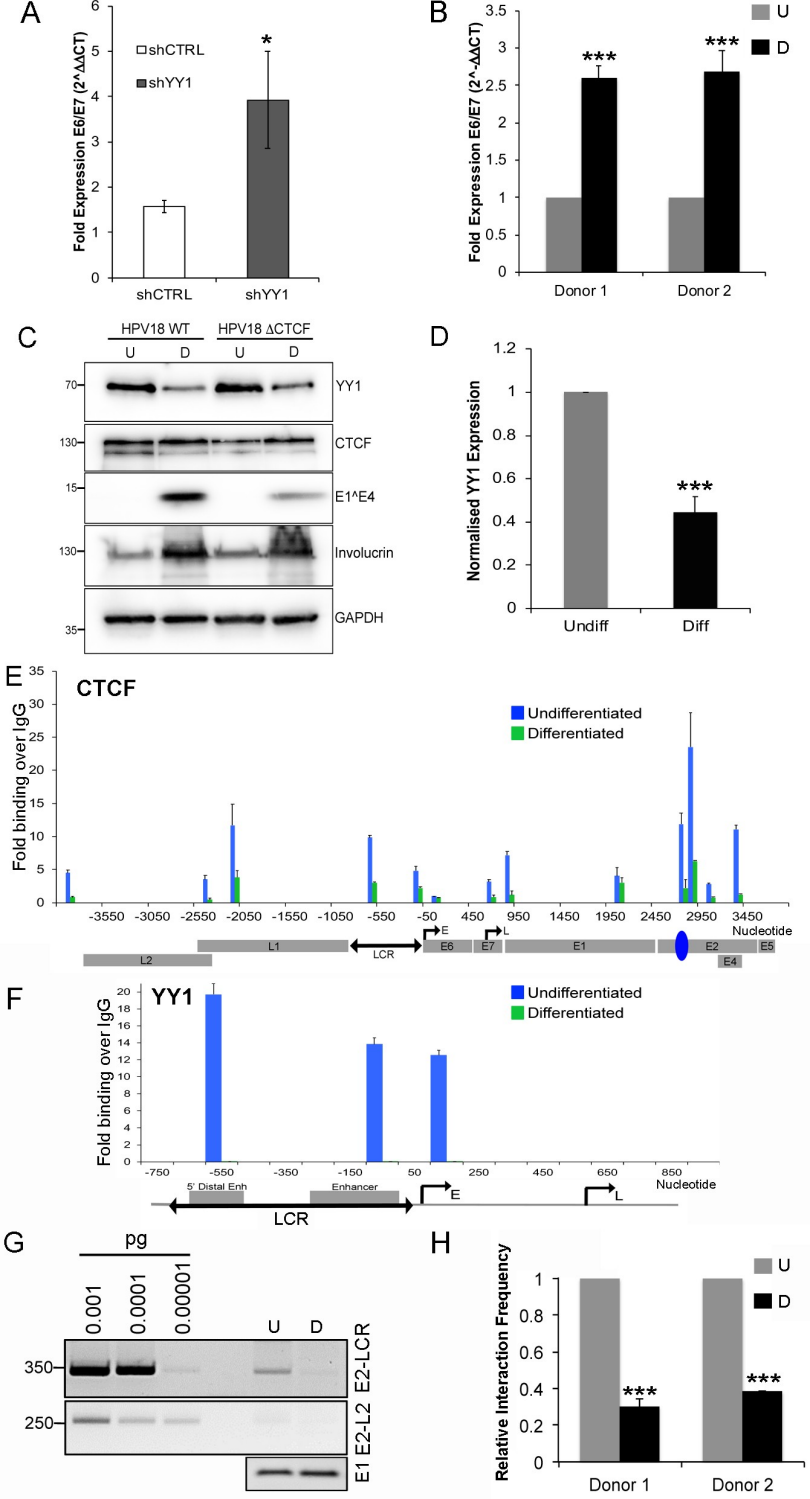
Host cell differentiation controls E2–LCR intrachromosomal interactions through repression of YY1 protein expression. **(A)** Fold expression change of E6/E7 encoding transcripts was determined by qRT-PCR following shYY1 normalised to β-actin and compared to shCNT. Data show mean ± SEM of three independent experiments performed in triplicate. (**B**) Fold expression of E6/E7 encoding transcripts after methylcellulose-induced keratinocyte differentiation was determined by qRT-PCR. Data show mean ± SEM in two keratinocyte donors normalised to β-actin and compared to undifferentiated cells. (**C**) YY1 protein expression in undifferentiated and differentiated HPV18 WT and ΔCTCF HFKs was assessed by western blotting. Involucrin and HPV18 E1^E4 expression was analysed as markers of differentiation. **(D)** YY1 protein expression was quantified and normalised to GAPDH. Data show normalised YY1 expression relative to undifferentiated cells in four independent experiments ± SEM. CTCF (**E**) and YY1 (**F**) recruitment in undifferentiated and differentiated cells were assessed by ChIP-qPCR. Data are represented as mean ± SD. **(G)** E2–LCR interactions were determined by 3C analysis in undifferentiated and differentiated keratinocytes. The top panel shows amplification of E2–LCR ligation products, and the middle panel shows amplification of E2–L2 ligation products. Lower panel shows E1 ORF amplification. **(H)** E2–LCR interactions in HPV18 WT genomes were quantified in multiple experimental repeats in each of two independent donors following cellular differentiation. Data are shown as mean ± SEM. **p < 0.01, ***p < 0.001 in all experiments. All raw quantitative data are enclosed in S1 Data. ChIP-qPCR, chromatin immunoprecipitation followed by quantitative PCR; CTCF, CCCTC-binding factor; D, differentiated; E, early promoter; GAPDH, Glyceraldehyde-3-phosphate dehydrogenase; HFK, human foreskin keratinocyte; HPV, human papillomavirus; IgG, immunoglobulin G; L, late promoter; LCR, long control region; ORF, open reading frame; qRT-PCR, quantitative reverse transcriptase PCR; SEM, standard error of the mean; shCNT, nontargeting shRNA control; shRNA, short hairpin RNA; shYY1, shRNA depletion of YY1; U, undifferentiated; WT, wild-type; YY1, Yin Yang 1; 3C, chromosome conformation capture.

It has previously been shown using transcriptional reporter plasmids that YY1 plays a pivotal role in the repression of HPV enhancer activity [7,8]. To confirm that YY1 is an essential repressor of HPV18 early gene expression in the context of the HPV genome, YY1 protein was depleted by YY1-specific shRNA expression as previously described and E6/E7 encoding viral transcripts quantified by qRT-PCR. Depletion of YY1 resulted in an over 20-fold increase in E6/E7 transcript levels (Fig 6A), confirming the role of YY1 as a transcriptional repressor in the HPV life cycle.

HPV gene expression during the virus life cycle is dependent on keratinocyte differentiation. Differentiation of infected keratinocytes in the midlayers of epithelia corresponds to an increase in early promoter activity [34–37]. In agreement with these studies, synchronous differentiation of keratinocytes by suspension of keratinocytes in semisolid medium for 48 hr resulted in a 2.6-fold increase in E6/E7 encoding transcripts in both keratinocyte donors (Fig 6B) and protein expression of the intermediate–early keratinocyte differentiation marker involucrin and a marker of the productive phase of the HPV life cycle, E1^E4 (Fig 6C). It has previously been shown that CTCF protein is localised to the nucleus of human keratinocytes and that expression is reduced in differentiated layers of human skin [38] and following morphological differentiation of human corneal epithelial cells [39]. Because of the known interaction between CTCF and YY1 and the functional role of this interaction in 3D chromatin loop formation, we also analysed YY1 expression in HPV18-genome–containing keratinocytes grown in monolayer (undifferentiated) or synchronously differentiated by suspension in methylcellulose for 48 hr. Western blot analysis of lysates of undifferentiated and differentiated cells revealed no difference in CTCF protein expression (Fig 6C), and importantly, CTCF protein was expressed at similar levels in HPV18 WT- and ΔCTCF-genome–containing cells (expression in HPV18 ΔCTCF compared to WT was 0.94-fold ± 0.18 SD; *p* = 0.97). However, a significant reduction of YY1 protein expression was observed in both HPV18 WT- and ΔCTCF-genome–containing cultures (Fig 6C and 6D), and this was consistent in two independent keratinocyte donors.

Since keratinocyte differentiation results in a marked reduction in YY1 protein expression, we examined whether cellular differentiation results in reduced CTCF and YY1 recruitment to the HPV18 genome. HPV18 WT-genome–containing cells were differentiated in methylcellulose and CTCF, and YY1 recruitment was analysed by ChIP. Differentiation of the cells resulted in reduced CTCF recruitment throughout the viral genome (Fig 6E) and a dramatic and complete loss of YY1 recruitment to the viral LCR (Fig 6F). To determine the effect of the differentiation-induced reduction in YY1 expression and recruitment to the HPV18 genome on the interaction between the E2–CTCF binding site and the viral LCR, 3C analysis was carried out using undifferentiated HPV18 WT-genome–containing cells harvested after growth in monolayer culture or genome-containing cells differentiated through incubation in methylcellulose for 48 hrs. We found that E2–LCR interactions were significantly reduced following cellular differentiation in two independent keratinocyte donors (Fig 6G and 6H).

### Host cell differentiation results in epigenetic de-repression of HPV18 chromatin and increased LCR accessibility

**Fig 7:**
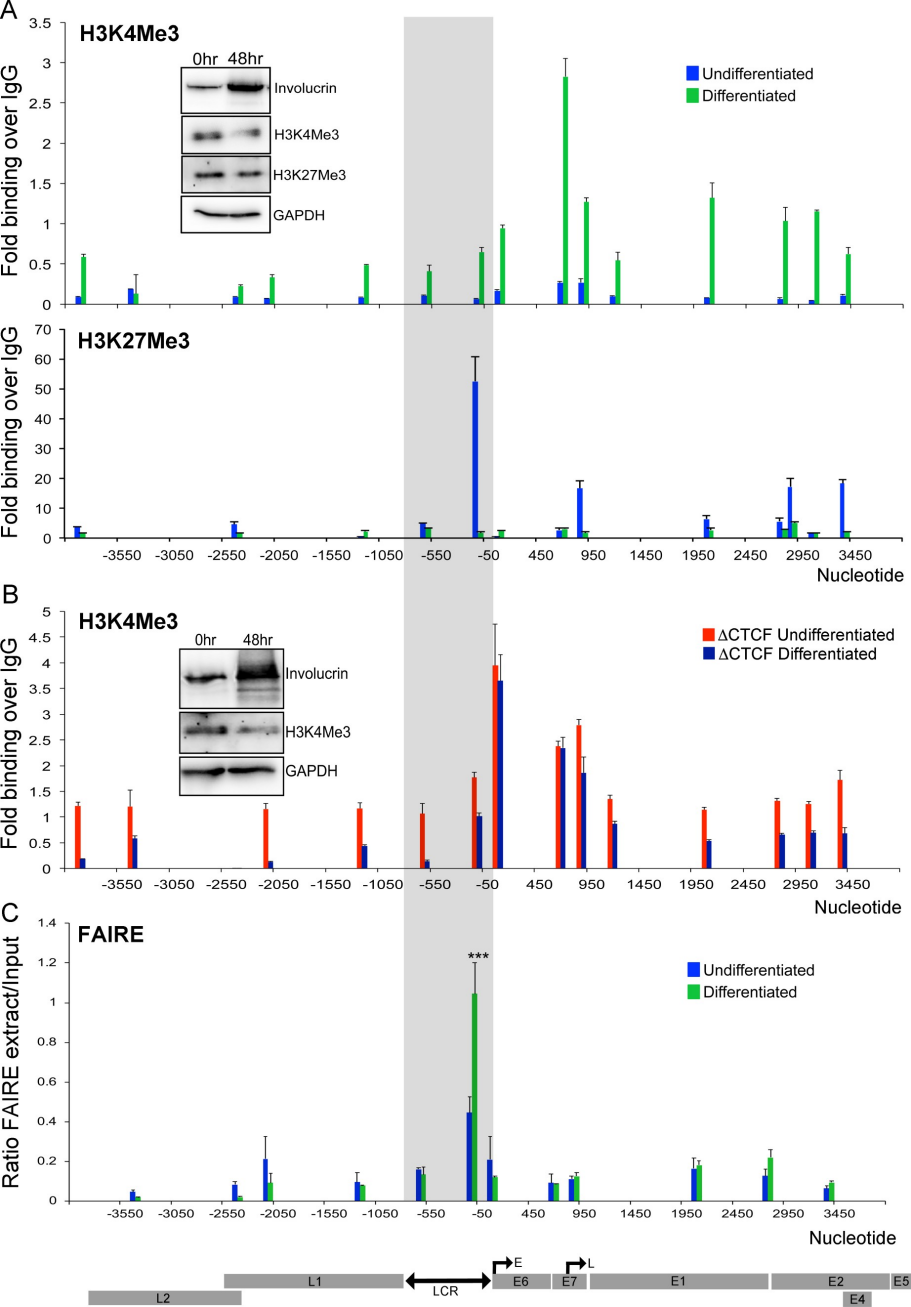
Host cell differentiation induces epigenetic switching and enhancer accessibility in HPV18 episomes. **(A)** Enrichment of H3K4Me3 (upper panel) and H3K27Me3 (lower panel) epigenetic marks was assessed in HPV18 episomes in undifferentiated (blue) and methylcellulose-differentiated (green) cells. Data show qPCR analysis at defined regions in the HPV18 genome as fold binding over IgG. The inset western blots show relative protein expression. **(B)** Enrichment of H3K4Me3 was assessed in ΔCTCF HPV18 episomes in undifferentiated (red) and methylcellulose-differentiated (purple) cells. The inset western blots show relative protein expression. (**C**) Chromatin accessibility following host cell differentiation was assessed by FAIRE. Data show qPCR analysis of amplicons in the HPV18 genome of FAIRE-extracted samples compared to Input in undifferentiated and differentiated HPV18 WT HFKs. The linearised HPV18 genome and CTCF–E2 ORF binding site are indicated. Data are represented as mean ± SD. (***p < 0.001). All raw quantitative data are enclosed in S1 Data. CTCF, CCCTC-binding factor; E, early promoter; FAIRE, formaldehyde-assisted isolation of regulatory elements; H3K4Me3, histone 3 lysine 4 trimethylation; H3K27Me3, histone 4 lysine 27 trimethylation; HPV, human papillomavirus; IgG, immunoglobulin G; L, late promoter; LCR, long control region; ORF, open reading frame; qPCR, quantitative PCR; WT, wild-type.

We next tested whether the decreased looping between the E2 ORF and the LCR we observed on keratinocyte differentiation resulted in changes in the epigenetic status and chromatin accessibility of HPV18 WT genomes. ChIP-qPCR analysis of H3K4Me3 and H3K27Me3 levels revealed enrichment of the active H3K4Me3 mark and loss of H3K27Me3 modifications, indicative of transcriptional de-repression following differentiation (Fig 7A). In contrast, differentiation of ΔCTCF HPV18-genome–containing cells that we previously showed were in enriched H3K4Me3 histone marks when undifferentiated (Fig 2B) did not result in any further enrichment of H3K4Me3 following differentiation, indicating aberrant regulation of epigenetic changes upon cellular differentiation in genomes unable to recruit CTCF (Fig 7B). We next examined whether this switching of epigenetic modifications results in increased accessibility of the chromatin using FAIRE. Our data demonstrated that differentiation of HPV18-genome–containing cells resulted in significant depletion of nucleosomes in the HPV18 LCR (Fig 7C). Taken together, our data demonstrate that upon cellular differentiation, reduced YY1 protein expression and recruitment to the viral LCR leads to the loss of chromatin loop formation, depletion of repressive epigenetic marks, and an associated increase in LCR chromatin accessibility. These observations therefore elucidate the mechanism underlying the progressive up-regulation of HPV18 E6 and E7 expression during keratinocyte differentiation, a mechanism likely to play a critical role in the successful completion of the viral life cycle.

## Discussion

**Fig 8:**
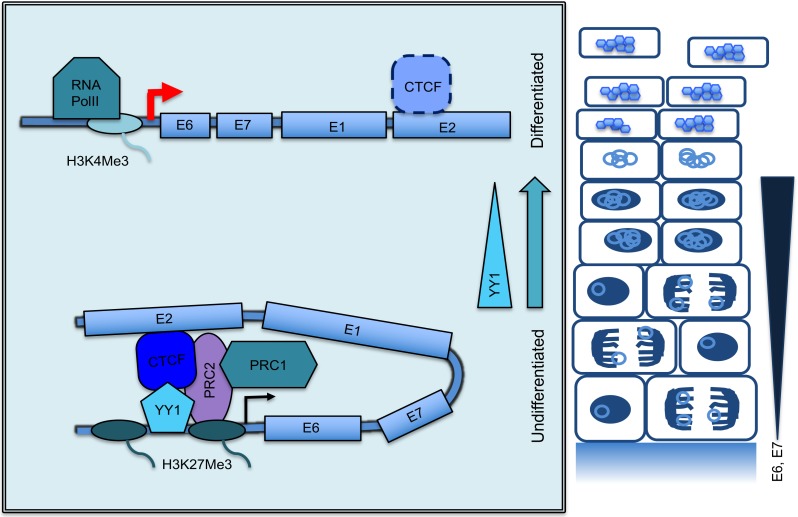
Model of differentiation-dependent looping and epigenetic control of HPV oncogene transcription. The HPV life cycle is dependent on infection of differentiating epithelia. Infection is established in the undifferentiated basal cells, in which a tightly controlled programme of viral gene expression is initiated. Expression of viral early genes (*E6*, *E7*) is attenuated in undifferentiated cells by the establishment of a CTCF-YY1–dependent chromatin loop of epigenetically repressed chromatin. As cells begin the programme of terminal differentiation, increased E6/E7 expression is required to maintain the cell in a proliferative state. This is coordinated by a differentiation-induced reduction in YY1 protein expression, which releases the repressive chromatin loop and attenuates PRC complex recruitment and loss of H3K27Me3 deposition. Increased accessibility and reduced H3K27Me3 at the viral enhancer results in de-repression of the early promoter, increased RNA Pol II recruitment, and H3K4Me3 deposition, resulting in increased *E6*/*E7* oncogene expression. CTCF, CCCTC-binding factor; H3K4Me3, histone 3 lysine 4 trimethylation; H3K27Me3, histone 3 lysine 27 trimethylation; HPV, human papillomavirus; PRC, polycomb repressor complex; RNA Pol II, RNA polymerase II; YY1, Yin Yang 1.

CTCF is a major regulator of host and virus transcription and mediates many of its functions by the coordination of dynamic long-range chromosomal interactions [15]. In this study, we show that mutation of the CTCF binding site with the E2 ORF of HPV18 results in a significant depletion of CTCF binding throughout the HPV genome. This interesting phenomenon suggested that intramolecular interactions occur between distinct regions of the HPV18 episome and that these interactions are stabilised by CTCF bound at the E2 ORF. For example, CTCF association with LCR-specific sequences, devoid of CTCF consensus binding sites [13], could occur via indirect interaction with the CTCF-bound E2 ORF, suggesting that the viral genome is organised by distinct intramolecular interactions. The global reduction of CTCF binding, by either mutation of the E2 binding site or by induction of CTCF-specific shRNA, resulted in increased E6/E7 transcript and protein production. We therefore hypothesised that CTCF mediates intrachromosomal interactions that are important for controlling the activity of the viral early promoter (depicted in Fig 8).

Analysis of the epigenetic status of the HPV18 WT episome revealed several important features. H3K4Me3 enrichment, indicative of active promoter regions, was observed at the early and late promoter regions in comparison to other regions in the HPV18 genome. In contrast, repressive H3K27Me3 levels were low at the early promoter, consistent with a previous study in HPV31 [10]. However, higher levels of H3K27Me3 were observed in the viral LCR, suggesting epigenetic repression of enhancer activity in undifferentiated cells, which has not previously been shown. Enhanced enrichment of H3K27Me3 was also observed at the late promoter. Quantitative analysis of these epigenetic marks in HPV18 ΔCTCF episomes demonstrated that attenuation of CTCF binding resulted in dramatic epigenetic switching in the viral genome, as evidenced by increased accessibility of the viral enhancer within the LCR and an enrichment of H3K4Me3 alongside a global loss of H3K27Me3 marks. This alteration in chromatin accessibility and epigenetic status correlated with enhanced recruitment of RNA Pol II. We previously reported that mutation of the CTCF binding within the E2 ORF of HPV18 results in a reduction in exon 416 to 929 inclusion [13]. Cotranscriptional splicing of RNA is physically linked to RNA Pol II activity, and it has been clearly demonstrated that transcription elongation dynamics influence intron identification and processing by the spliceosome (reviewed by [40]). We therefore hypothesise that the observed alteration of HPV transcript splicing [13] is due to altered RNA Pol II dynamics, and we will formally test this hypothesis in future studies.

YY1 in part functions as a cellular transcriptional repressor by mediating the recruitment of PRC1 and PRC2 to specific enhancer loci [41,42]. PRC2 catalyses H3K27Me3 deposition while PRC1 catalyses H2AK119Ub deposition, together resulting in transcriptional repression. Since a dramatic loss of H3K27Me3 enrichment was observed in HPV18 genomes unable to bind CTCF, we hypothesised that PRC2 was depleted. We demonstrated significant loss of the PRC2 component EED, providing evidence that PRC2 recruitment is reduced in HPV18 ΔCTCF genomes, resulting in reduced H3K27Me3 deposition. In addition, a significant reduction of the PRC1 catalytic subunit Ring1B to the viral early promoter was observed, resulting in reduced H2AK119Ub deposition. The reduction in both PRC1 and PRC2 recruitment to the viral LCR following abrogation of CTCF binding explains the loss of repressive epigenetic marks and increased chromatin accessibility and activity of the P_105_ early promoter.

CTCF and YY1 can physically associate to stabilise chromatin loops, and organisation of the host cell genome in this manner controls specific gene-expression switching in X chromosome inactivation and neural cell differentiation [32,33]. Combined with our data showing the global loss of CTCF recruitment within the HPV18 genome when the dominant E2 ORF binding site was mutated and CTCF-mediated regulation of LCR topology and YY1 enrichment, we hypothesised that CTCF and YY1 mediate an intramolecular interaction between the E2 ORF and the LCR to stabilise an epigenetically repressed chromatin domain. We demonstrated a specific interaction between the E2 ORF and LCR that was dependent on CTCF and YY1 expression. We show that disruption of the E2–LCR interaction results in transcriptional de-repression of the viral early promoter through a dramatic alteration of the epigenetic status of the viral episome and increased chromatin accessibility. Such short-range interactions have been previously identified in cellular loci using similar methods, including interactions between the insulin gene promoter and distal enhancer and within the 2.5 kbp CD68 gene [43,44]. In addition, a recent study has demonstrated genomic interactions within the KSHV genome, ranging from 5 kbp to >80 kbp in size [45]. These studies provide evidence that short-range genomic loci are important in the regulation of host cell and episomal virus transcription regulation. However, it is important to note that the 3C analysis used in our studies could also detect interchromosomal interactions between multiple viral episomes in the same cell. Such interactions between viral episomes could stabilise the formation of viral super enhancers [46] and/or function in the homologous recombination-dependent replication of episomes in replication centres [47].

Our results provide important insight into YY1 and CTCF function in transcriptional control. We demonstrate that depletion of CTCF reduces YY1 recruitment and vice versa, suggesting that CTCF and YY1 bind to the viral genome in a cooperative manner. CTCF and YY1 have been shown to physically interact [33], and studies have shown that YY1 and CTCF can anchor loops via homo- and heterodimerisation [32,48]. It has previously been shown that over 30% of YY1-occupied sites in the human genome are at locations directly adjacent to CTCF-occupied sites, but that the binding of these factors do not directly colocalise, suggesting that these factors work together to cooperatively influence occupancy at adjacent binding sites [32, 49]. YY1 is enriched at sites within the host chromatin that engage in 3D looping, and YY1 enrichment at these sites is reduced when these elements are not connected, suggesting that YY1 binding is stabilised by 3D chromatin interactions [32]. It has therefore been suggested that CTCF binding initially serves as an architectural ‘seed’ and that YY1 binding then connects CTCF-bound nearby regulated genes and enhancers. Our results show that depletion of CTCF reduces YY1 recruitment, consistent with this hypothesis, but also suggest that CTCF binding is also influenced by YY1 recruitment.

In the HPV life cycle, the E6 and E7 oncoproteins are expressed at low levels in basal keratinocytes, presumably to limit host immune activation and because their combined functions in the cell cycle to maintain expression of the cellular DNA replication machinery are less important in these undifferentiated, cycling cells. Host cell differentiation is associated with increased viral early transcript production, resulting in increased E6/E7 protein expression as well as activation of the late promoter [35]. E6 and E7 act to maintain host cell proliferation, maintaining viral access to the host cell DNA replication machinery, and it has been shown that viral genome amplification in differentiated epithelia requires robust E6 expression [50]. Our data demonstrated a significant reduction in E2–LCR loop formation in differentiated keratinocytes and an associated reduction in epigenetic repression of the viral genome and increased accessibility of the LCR. Our results support a model in which the level of YY1 protein expression controls viral oncogene expression during differentiation.

The in-depth analysis of the epigenetic status and topology of HPV18 episomes in a physiological model of the HPV18 life cycle has provided mechanistic insight into the underlying differentiation-dependent control of HPV18 early gene expression. We have demonstrated that CTCF and YY1 together function in coordinating a transcriptional switch that is directly linked to host cell differentiation (Fig 8). This ensures low-level expression of viral proteins in the basal cells, which presumably facilitates persistence in vivo. As infected cells differentiate, the epigenetically repressed chromatin loop responsible for attenuating activity of the viral enhancer is disrupted, and the repressive epigenetic marks are lost. We show that this mechanism of controlling viral gene expression is regulated by CTCF-YY1 interactions within the HPV18 episome; as cells differentiate, YY1 protein expression is repressed and loop formation is disrupted, promoting enhancer activation.

Whether this mechanism of differentiation-dependent regulation of HPV oncogene expression plays a role in HPV-driven cancer is not clear, but it is tempting to speculate that this is the case. YY1 binding sites are often mutated in the HPV16 genome in cervical cancer [51,52], and a recent study has demonstrated that an open chromatin state of the viral LCR correlates with high E6/E7 expression in a model of HPV16-driven carcinogenesis [12]. Since the CTCF binding site within the E2 ORF is conserved in HPV16 [13], we predict that a similar mechanism of oncogene repression exists in HPV16. In addition, CTCF recruitment to the E2 ORF within integrated HPV18 DNA in HeLa cells is very low even though the CTCF binding site is intact [53]. Low CTCF binding in HeLa cells is coincident with low H3K27Me3 and high H3K4Me3 marks at the viral LCR and early promoter, combined with high E6/E7 transcript production, which is in agreement with our findings in HPV18 ΔCTCF episomes. It will therefore be of importance to determine whether CTCF-mediated attenuation of viral oncogene expression is disrupted in HPV-driven cancers. To begin to answer this question, we have analysed CTCF binding-site mutations in a cohort of 3,215 HPV16 positive lesions and correlated our findings with clinical outcome [54]. A variation in the CTCF binding-site motif 2 was discovered in 357 individual HPV16 sequences (A^2938^ to G), which we predict would enhance CTCF binding [55]. Interestingly, the presence of this genetic variation in the HPV16 genome is significantly associated with decreased cancer incidence when compared to the cases with no variation in the vicinity of the binding site (*p* = 0.050, one-tailed Fisher’s test). We therefore speculate that in lesions that contain this variant of HPV16, CTCF may bind with higher affinity and have a more significant effect on the attenuation of E6/E7 expression, thereby reducing the risk of cancer development. In addition to genetic variations within the CTCF binding site, we also found many sequences that had no sequence information at and around the E2 ORF CTCF binding site. This could be due to integration of the virus such that the E2 coding sequence is disrupted, but in addition to this widely reported mechanism of HPV-driven carcinogenesis, it will be important to determine the mechanism and consequence of CTCF exclusion in cancers with integrated HPV DNA.

## Materials and methods

### Ethics statement

The collection of circumcised foreskin tissue from newborns for the isolation of primary HFKs for investigation of HPV biology was approved by Southampton and South West Hampshire Research Ethics Committee A (REC Reference number 06/Q1702/45). Written consent was obtained from the parent or guardian. The study was approved by the University of Birmingham Ethical Review process (ERN_16–0540).

### Plasmids and antibodies

pGEMII-HPV18 (gift from F. Stubenrauch, University of Tübingen, Germany) contains the complete HPV18 genome cloned into the *Eco*RI site of pGEMII and was used to create pGEMII-HPV18-ΔCTCF, which contains three conservative nucleotide substitutions (C^2993^T, G^3005^A, T^3020^C) within the E2 coding region to abolish CTCF binding as previously described [13].

CTCF (61311), H3K4Me3 (39915), H3K27Me3 (39155), RNA Pol II (61081), SP1 (39058), TEF1 (61644), EZH2 (39901), EED (61203), and Ring1B (39663) and antibodies were purchased from Active Motif (La Hulpe, Belgium). YY1 antibody (SC-7341X) was purchased from Santa Cruz Biotechnology (Dallas, TX, United States of America). H2AK119Ub (D27C4) was purchased from Cell Signaling Technology, Inc (Danvers, MA, USA). FLAG M2 was purchased from Sigma-Aldrich (Gillingham, United Kingdom), E7 clone 8E2 (ab100953) was purchased from Abcam (Cambridge, UK), and E6 clone G7 (SC-365089) and Glyceraldehyde-3-phosphate dehydrogenase (GAPDH) antibody were purchased from Santa Cruz Biotechnology (Dallas, TX, USA). Monoclonal HPV18 E1^E4 antibody 1D11 was produced by S. Roberts [56]. All horseradish-peroxidase–conjugated secondary antibodies were purchased from Jackson Laboratories (Bar Harbor, ME, USA).

### Primary keratinocyte culture, transfection, and organotypic raft culture

The transfection of normal primary HFKs from neonatal foreskin epithelia with recircularised HPV18 WT and ΔCTCF genomes was performed in S. Roberts’ laboratory by J. Parish as previously described [13,57]. To eliminate donor-specific effects, primary cells from two foreskin donors were used: one isolated in house and one commercially available (Lonza, Basel, Switzerland). Episome copy number in each cell line was determined by Southern blotting as described previously [26] and calculated by densitometry of three technical repetitions of each HPV18-transfected keratinocyte donor compared to the 50 copies per cell loading control. Digestion of the DNA with *Eco*RI results in linearisation of episomes, whereas digestion with *Bgl*II restricts the host cell DNA but not the viral DNA to reveal integrated or multimeric virus. *Dpn*I digests input DNA only.

Organotypic raft cultures were prepared as previously described [13,57,58] and cultured for 14 d in E medium [58] without epidermal growth factor to allow cellular stratification. Rafts were fixed in 3.7% formaldehyde and paraffin embedded prior to sectioning (Propath Ltd., Hereford, UK).

### Methylcellulose-induced differentiation of keratinocytes

HPV18-genome–containing keratinocytes (3 × 10^6^ cells) were suspended in E medium containing 10% FBS and 1.5% methylcellulose and incubated at 37°C, 5% CO_2_ for 48 h. Cells were then harvested by centrifugation at 250 × *g* and then thoroughly washed with ice-cold PBS. Cells were then either resuspended in medium containing 1% formaldehyde to cross-link for ChIP and 3C or in urea lysis buffer for protein extraction.

### Chromatin immunoprecipitation

ChIP assays were carried out using the ChIP-IT Express kit (Active Motif) following the manufacturer’s instructions. Briefly, cells were fixed in 1% formaldehyde for 3 min at room temperature, quenched in 0.25 M glycine, and washed in ice-cold PBS. Nuclei were released by 40 strokes in a tight dounce homogeniser. Samples were sonicated at 25% amplitude for 30 s on/30 s off for a total of 15 min using a Sonics Vibracell sonicator fitted with a microprobe. ChIP efficiency was assessed by quantitative PCR (qPCR) using SensiMix SYBR master mix using an MXPro 3000 (Agilent Technologies, Santa Clara, CA, USA). Primer sequences for ChIP experiments are shown in Table 1. Cycle threshold (C_T_) values were calculated at a constant threshold for each experiment, and fold-enrichment–compared to negative control FLAG antibody was calculated using the following formula:

Fold binding over IgG = (2^ΔCT target^)/(2^ΔCT IgG^),

where ΔC_T_ target = Input C_T_−Target C_T_ and ΔC_T_ IgG = Input C_T_−IgG C_T_. Each ChIP experiment was performed in triplicate, and data shown are the mean ± SD of a representative experiment. Biological repeats were performed for each experiment a minimum of three times with similar results.

**Table 1:** Primer pairs used for ChIP and FAIRE analysis of HPV18 genomes.

Amplicon midpoint*	Forward Primer (5′-3′)	Reverse Primer (5′-3′)
–3,886	TATGTGTGCTGCCATGTCCC	CTGTGGCAGGGGACGTTATT
–3,352	GGGGTCGTACAGGGTACATT	GATGTTATATCAAACCCAGACGTG
–2,384	TCTGCCTCTTCCTATAGTAATGTAACG	GGAATAAAATAATATAATGGCCACAAA
–2,092	CCTCCTTCTGTGGCAAGAGT	GGTCAGGTAACTGCACCCTAA
–1,198	AGTCTCCTGTACCTGGGCAA	AACACCAAAGTTCCAATCCTCT
–556	GTGTGTTATGTGGTTGCGCC	GGATGCTGTAAGGTGTGCAG
–57	ACTTTCATGTCCAACATTCTGTCT	ATGTGCTGCCCAACCTATTT
155	TGTGCACGGAACTGAACACT	CAGCATGCGGTATACTGTCTC
751	CGAACCACAACGTCACACAAT	ACGGACACACAAAGGACAGG
943	AGTGTGAAGCCAGAATTGAGC	ACCACGGACACACAAAGGA
1,500	GCAATGTATGTAGTGGCGGC	TACACTGCTGTTGTTGCCCT
2,200	TTATAGGCGAGCCCAAAAAC	CCAATCTCCCCCTTCATCTAT
2,819	TGCAGACACCGAAGGAAACC	CATTTTCCCAACGTATTAGTTGCC
2,989	GGCAACTAATACGTTGGGAAAA	TGTCTTGCAGTGTCCAATCC
3,165	AGGTGGCCAAACAGTACAAGT	GCCGTTTTGTCCCATGTTCC
3,488	TGGGAAGTACATTTTGGGAATAA	TCCACAGTGTCCAGGTCGT

All primer pairs generated a single product as determined by dissociation curve analysis.

*Primer midpoint is stated relative to the 7857/1 nucleotide position on the HPV18 episome (Genbank: AY262282.1).

**Abbreviations:** ChIP, chromatin immunoprecipitation; FAIRE, formaldehyde-assisted isolation of regulatory elements; HPV, human papillomavirus.

### Western blotting

Cells were lysed in urea lysis buffer (8 M Urea, 100 mM Tris-HCl, pH 7.4, 14 mM β-mercaptoethanol, protease inhibitors) and protein concentration determined by Bradford assay. Equal amounts of protein were separated by SDS-PAGE and western blotting carried out using conventional methods.

### RNA-Seq and qRT-PCR

RNA was extracted with an RNeasy Mini Kit (Qiagen, Hilden, Germany) according to the manufacturer’s protocol and DNase treated. For RNA-Seq, libraries were prepared using TruSeq Stranded mRNA Library Prep kit for NeoPrep (Illumina, San Diego, CA, USA) using 100 ng total RNA input according to manufacturer’s instructions. Libraries were pooled and run as 75-cycle–pair end reads on a NextSeq 550 (Illumina) using a high-output flow cell.

cDNA was synthesised using Superscript III (Invitrogen, Carlsbad, CA, USA) according to the manufacturer’s instructions. qPCR was performed using a Stratagene Mx3005P detection system with SyBr Green incorporation and the primers listed in Table 2.

**Table 2:** Primer sequences for qRT-PCR transcript analysis.

Primer Pair	Forward Sequence (5′-3′)	Reverse Sequence (5′-3′)
**E6/E7 (121–295)**	ATCCAACACGGCGACCCTAC	GCAGCATGCGGTATACTGTCTCTA
**E6 (131–180)**	GCGACCCTACAAGCTACCTG	GCAGTGAAGTGTTCAGTTCCG
**CTCF (exon 5–6)**	ATGTGCGATTACGCCAGTGTA	TGAAACGGACGCTCTCCAGTA
**β-actin**	GCTGTGCTATCCCTGTACGC	CAGGAAGGAAGGCTGGAAGA

**Abbreviations:** CTCF, CCCTC-binding factor; qRT-PCR, quantitative reverse transcriptase-PCR.

### FAIRE

Cells were fixed and chromatin extracted and sheared as described above for ChIP. FAIRE analysis was then carried out as previously described [29]. Briefly, two aliquots of chromatin were taken, each containing chromatin from approximately 2 × 10^6^ cells, one for Input and one for FAIRE. To the FAIRE samples, 150 μl water was added. To the Input sample, 150 μl water and 10 μl of 5 M NaCl were added, and the samples were incubated at 95°C for 15 min to reverse the crosslinks. RNaseA (10 μg/μl) was added, and the samples incubated at 37°C for 15 min. Proteinase K (0.5 μg/μl) was added followed by incubation at 67°C for 15 min.

Both Input and FAIRE samples were then extracted with 200 μl phenol:chloroform:isoamylalcohol (25:24:1) and the aqueous layer retained. DNA was precipitated by conventional methods and the pellet resuspended in 50–150 μl 50 mM Tris-HCl, pH 7.4, 10 mM EDTA. Recovery of FAIRE-extracted DNA in comparison to Input DNA was then determined by qPCR using the ΔΔC_T_ method. Primer sequences are shown in Table 1.

### 3C

A total of 1–1.5 × 10^7^ cells were trypsinised and resuspended in 1 ml 10% (v/v) FCS/PBS. Cells were passed through a 70 μm cell strainer and 9.5 ml of 1% formaldehyde in 10% FCS/PBS added before incubation for 10 min at RT with end-to-end rotation. Glycine was added to a final concentration of 125 μM before the cells were pelleted at 4°C. Cells were resuspended in 5 ml of ice cold lysis buffer (10 mM Tris-HCl, pH 7.7, 10 mM NaCl, 5 mM MgCl_2_, 0.1 mM EGTA, protease inhibitors) and incubated on ice for 10 min. Samples were centrifuged at 400 × *g* for 5 min at 4°C to pellet the nuclei. Five hundred μl 1.2× restriction enzyme buffer and 0.3% (final concentration) SDS were added and samples incubated at 37°C for 1 hr while shaking at 900 rpm. Fifty μl of 20% Triton X-100 was then added, followed by incubation at 37°C for 1 hr with shaking at 900 rpm. Prior to digestion, an aliquot was removed for assessment of digestion efficiency. Four hundred units of *Nla*III restriction enzyme were added, and the samples were incubated at 37°C overnight with shaking at 900 rpm. An aliquot of each sample was removed and assessment of digestion efficiency performed by adding 500 μl of 5 mM EDTA, pH 8.0, 10 mM Tris-HCl, pH 8.0, 0.5% SDS, and 20 μg proteinase K and incubating at 65°C for 30 min. One μg RNase A was added, followed by incubation at 37°C for 2 hr. The DNA was extracted with PCI and ethanol precipitated using conventional methods and the pellet resuspended in 60 μl dH_2_0. Digestion efficiency of the viral genomes was assessed by qPCR of genome regions sensitive and insensitive to containing *Nla*III restriction sites (Table 3) and comparison of C_T_ values as described in [59].

**Table 3:** Primers used for 3C analysis of HPV18 genomes.

Primer Pair	Forward Primer (5′-3′)	Reverse Primer (5′-3′)
E2–CTCF (*)	N/A	CTTGCAGTGTCCAATCCTCG
**LCR–YY1 (1)**	N/A	GGTGCAGCATCCTTTTGACA
**L2 (2)**	N/A	CCCCGTACCAGAAGAAGCAA
**E1 (3 and 4)**	AGTGTGAAGCCAGAATTGAGC	ACCACGGACACACAAAGGA
**HPV18 Digest efficiency**	GCAAACGGGCTTCGGTAACT	TTATCTGCTAACGTGGTGCCC

**Abbreviations:** CTCF, CCCTC-binding factor; HPV, human papillomavirus; LCR, long control region; YY1, Yin Yang 1; 3C, chromosome conformation capture.

For ligation, 40 μl of 20% SDS was added to the samples, followed by incubation for 25 min at 65°C with shaking at 900 rpm. A total of 6.125 ml 1.15× ligation buffer and 1% (final concentration) Triton X-100 was added. Samples were incubated for 1 hr at 37°C with gentle shaking. One hundred units of T4 DNA ligase were added and the samples incubated for 4 hr at 16°C, followed by 30 min at RT. Three hundred μg proteinase K was added, and the samples were incubated at 65°C overnight.

To purify the digested DNA, 300 μg RNase A was added and the samples incubated for 45 min at 37°C. The DNA was extracted with PCI twice and ethanol precipitated. Finally, the DNA pellet was resuspended in 10 mM Tris-HCl, pH 7.5. Ligation of specific regions of the HPV18 genome was assessed by PCR using sense primers specific to the L1 and E2 ORFs to detect interactions between the CTCF-bound E2 ORF and the viral LCR and sense primers specific for the L2 and E2 ORFs to detect ligation events that could occur by chance (Table 3). PCR products were assessed by agarose gel electrophoresis and compared to products obtained with a synthesised DNA template equivalent to the predicted ligation product (GeneStrings). Products were sequenced and quantified with a Fusion FX imaging system.

### Lentiviral transduction of keratinocytes and shRNA expression

Human embryonic kidney 293T (HEK293T) cells were grown in Dulbecco’s modified Eagle’s medium (DMEM) supplemented with 10% FBS and transfected with second-generation lentiviral packaging plasmids, psPAX2 and pMD2.G, and pTRIPZ-shRNA expressing plasmid using polyethylenimine (PEI) Max at a DNA-to-reagent ratio of 1:3. Medium containing lentiviral particles was recovered at 48 and 72 hr post transfection and passed through a 0.45 μM filter. The resulting recombinant lentiviruses were concentrated using Vivaspin ultrafiltration spin columns (50,000 MWCO PES) and used to spin infect HPV18-genome–containing keratinocytes growing in 6-well plates in E medium containing 8 μg/ml polybrene after feeders had been removed. Plates were spun at 3,220 × *g* for 90 min to facilitate infection, after which media were replaced with E medium. Twenty-four hr later, lentiviral infection was repeated as described above before cells were detached and seeded onto fresh irradiated feeder cells in 10 cm dishes. Puromycin to a final concentration of 1 μg/ml was added to the cells 72 hr later to select infected cells. shRNA expression was induced with 1 μg/ml doxycycline for 48 hr.

## Supporting information

S1 Table Quantitative analysis of RNA-Seq data showing CTCF transcript counts at the indicated exons expressed as counts per million reads. *Calculated compared to untransfected isogenic donor. CTCF, CCCTC-binding factor; RNA-Seq, RNA-Sequencing.
(DOCX)

S2 Table Quantitative analysis of RNA-Seq data showing HPV18 transcripts counts at the indicated ORF positions expressed as counts per million reads. *ΔCTCF HPV18 gene expression compared to WT HPV18. CTCF, CCCTC-binding factor; HPV, human papillomavirus; ORF, open reading frame; RNA-Seq, RNA-Sequencing.
(DOCX)

S1 Fig Sequence of E2 ORF–LCR 3C ligation product. Showing 3C forward and reverse primer sites (red), *Nla*III digestions site (green), and CTCF binding site (blue). CTCF, CCCTC-binding factor; LCR, long control region; ORF, open reading frame; 3C, chromosome conformation capture.
(DOCX)

S1 Data Spreadsheet containing raw data files used for the generation of quantitative data contained within the manuscript. (XLSX)

## Abbreviations

bp: basepair
ChIP-qPCR: chromatin immunoprecipitation followed by quantitative PCR
CNT: nontargeting control
CTCF: CCCTC-binding factor
DMEM: Dulbecco’s modified Eagle’s medium
EBV: Epstein-Barr virus
EED: embryonic ectoderm development
Enh: enhancer
FAIRE: formaldehyde-assisted isolation of regulatory elements
GAPDH: Glyceraldehyde-3-phosphate dehydrogenase
H2AK119Ub: histone 2A lysine 119 ubiquitinylation
H3K27Me3: histone 3 lysine 27 trimethylation
H3K4Me3: histone H4 lysine 4 trimethylation
HFK: human foreskin keratinocyte
HPV: human papillomavirus
HSV-1: herpes simplex virus type 1
HVS: herpesvirus saimiri
IgG: immunoglobulin G
KSHV: Kaposi sarcoma–associated herpesvirus
LCR: long control region
M/I: multimeric/integrated
nt: nucleotide
OC: open circle
ORF: open reading frame
PEI: polyethylenimine
PRC: polycomb repressor complex
qPCR: quantitative PCR
qRT-PCR: quantitative reverse transcriptase-PCR
Ring1B: ring finger protein 1B
RNA Pol II: RNA polymerase II
RNA-Seq: RNA-Sequencing
SEM: standard error of the mean
shCNT: nontargeting shRNA control
shRNA: short hairpin RNA
shYY1: shRNA depletion of YY1
TFIID: transcription factor II D
WT: wild-type
YY1: Yin Yang 1
3C: chromosome conformation capture
