# Supplementary material for: Disruption of CTCF-YY1–dependent looping of the human papillomavirus genome activates differentiation-induced viral oncogene transcription
Source: PLoS Biol. 2018 Oct 25;16(10):e2005752. doi: 10.1371/journal.pbio.2005752 (PMC6219814; doi:10.1371/journal.pbio.2005752)
Supplement: S1 Table — *Calculated compared to untransfected isogenic donor. CTCF, CCCTC-binding factor; RNA-Seq, RNA-Sequencing. (DOCX) [file pbio.2005752.s001.docx]

| Exon | Reads per Million (RPM) | | | % Change* | | |
| --- | --- | --- | --- | --- | --- | --- |
|  | Untransfected | WT HPV18 | ΔCTCF HPV18 | WT | | ΔCTCF |
| 2 | 3.98 | 3.75 | 4.22 | -5.8% | + 6.0% | |
| 7 | 3.53 | 3.51 | 3.82 | -0.6% | + 8.2% | |
| 9 | 4.17 | 3.84 | 4.35 | -7.9% | + 4.3% | |
|  |  |  |  |  | | |
