# Supplementary material for: Disruption of CTCF-YY1–dependent looping of the human papillomavirus genome activates differentiation-induced viral oncogene transcription
Source: PLoS Biol. 2018 Oct 25;16(10):e2005752. doi: 10.1371/journal.pbio.2005752 (PMC6219814; doi:10.1371/journal.pbio.2005752)
Supplement: S2 Table — *ΔCTCF HPV18 gene expression compared to WT HPV18. CTCF, CCCTC-binding factor; HPV, human papillomavirus; ORF, open reading frame; RNA-Seq, RNA-Sequencing. (DOCX) [file pbio.2005752.s002.docx]

| Nucleotide position | ORF | Reads per Million (RPM) | | % Change* |
| --- | --- | --- | --- | --- |
|  |  | WT HPV18 | ΔCTCF HPV18 |  |
| 233 | E6 (upstream of splice) | 123.7 | 182.2 | + 47.3 % |
| 245 | E6 (downstream of splice) | 22.2 | 30.7 | + 38.3 % |
| 590 | E7 TSS | 72.3 | 84.4 | + 16.7 % |
| 2817 | E2 TSS | 11.65 | 10.01 | - 14.1 % |
