# Supplementary material for: Disruption of CTCF-YY1–dependent looping of the human papillomavirus genome activates differentiation-induced viral oncogene transcription
Source: PLoS Biol. 2018 Oct 25;16(10):e2005752. doi: 10.1371/journal.pbio.2005752 (PMC6219814; doi:10.1371/journal.pbio.2005752)
Supplement: S1 Fig — Showing 3C forward and reverse primer sites (red), NlaIII digestions site (green), and CTCF binding site (blue). CTCF, CCCTC-binding factor; LCR, long control region; ORF, open reading frame; 3C, chromosome conformation capture. (DOCX) [file pbio.2005752.s003.docx]

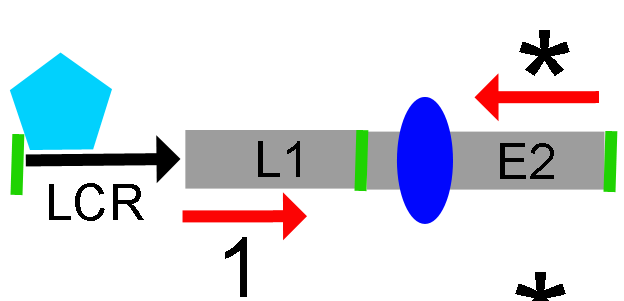


HPV18 3C L1-E2 ligation product

*

cttgcagtgtccaatcctcggttttgtatcgactttgtgcaaggccttgtagggccatttgcagttcaatagctttatgtgctttactttttgaaatgttataggctggcaccacctggtggtttaatgtctgtatgc[catg]ttgaggaatatgatttgcagtttatttttcagttgtgtactattactttaactgcagatgttatgtcctatattcatagtatgaatagcagtattttagaggattggaactttggtgttcccccccccccaactactagtttggtggatacatatcgttttgtacaatctgttgctattacctgtcaaaaggatgctgcacc

1

Forward primer (anchor primer *)

Reverse primer (primer 1)

*Nla*III digestion and ligation site

CTCF binding site
